# Quantitative monitoring of *Arabidopsis thaliana* growth and development using high-throughput plant phenotyping

**DOI:** 10.1038/sdata.2016.55

**Published:** 2016-08-16

**Authors:** Daniel Arend, Matthias Lange, Jean-Michel Pape, Kathleen Weigelt-Fischer, Fernando Arana-Ceballos, Ingo Mücke, Christian Klukas, Thomas Altmann, Uwe Scholz, Astrid Junker

**Affiliations:** 1 Leibniz Institute of Plant Genetics and Crop Plant Research (IPK) Gatersleben, Stadt Seeland D-06466, Germany

**Keywords:** Plant development, Research management, Arabidopsis thaliana, Imaging

## Abstract

With the implementation of novel automated, high throughput methods and facilities in the last years, plant phenomics has developed into a highly interdisciplinary research domain integrating biology, engineering and bioinformatics. Here we present a dataset of a non-invasive high throughput plant phenotyping experiment, which uses image- and image analysis- based approaches to monitor the growth and development of 484 *Arabidopsis thaliana* plants (thale cress). The result is a comprehensive dataset of images and extracted phenotypical features. Such datasets require detailed documentation, standardized description of experimental metadata as well as sustainable data storage and publication in order to ensure the reproducibility of experiments, data reuse and comparability among the scientific community. Therefore the here presented dataset has been annotated using the standardized ISA-Tab format and considering the recently published recommendations for the semantical description of plant phenotyping experiments.

## Background & Summary

Plants and plant-derived products provide an essential contribution to human nutrition and are the basis for food and feed. Societal and environmental challenges such as a fast growing human population and climate change force us to develop strategies for food security and to increase the productivity and resilience of agricultural systems. The growing demand for food and feed can only be tackled with the development of crop plants with higher biomass, increased nutrient and water efficiency as well as higher resistance to biotic and abiotic stressors. The prerequisite for this task is the deep understanding of plant growth and development as represented by the plant's ‘phenotype’ which refers to the entirety of features of a plant from the molecular, physiological to the architectural level. The plant’s phenotype (P) is the result of its genetic constitution (G) and the environmental conditions (E) that have been encountered during plant growth (P=G×E). Over the last decade the analysis of plant phenotypes has been increasingly approached by using high throughput phenotyping systems that enable the automated quantitative assessment of plant growth dynamics^1–3^. A variety of image-based methods and corresponding image analysis pipelines have been implemented in order to extract architectural and physiological plant parameters which are related to agronomically relevant traits such as yield and performance^4^.

At the *Leibniz Institute of Plant Genetics and Crop Plant Researc*h (IPK) Gatersleben, three high throughput plant phenotyping systems are installed which allow the automatic analysis of differently sized plants, from model to crop species. The LemnaTec systems for small, medium and large sized plants enable to grow and image up to 4608/520/1584 plants at a time with a maximal plant height up to 30/150/200 cm, respectively. All three installations are equipped with different camera sensor systems to measure responses by using the visible light (VIS), the near-infrared (NIR) spectrum and fluorescence imaging (FLUO). The systems follow the plant-to-sensor principle where plants are transported to the imaging chambers and watering/weighing stations in special carriers on conveyor belts.

**Figure 1:**
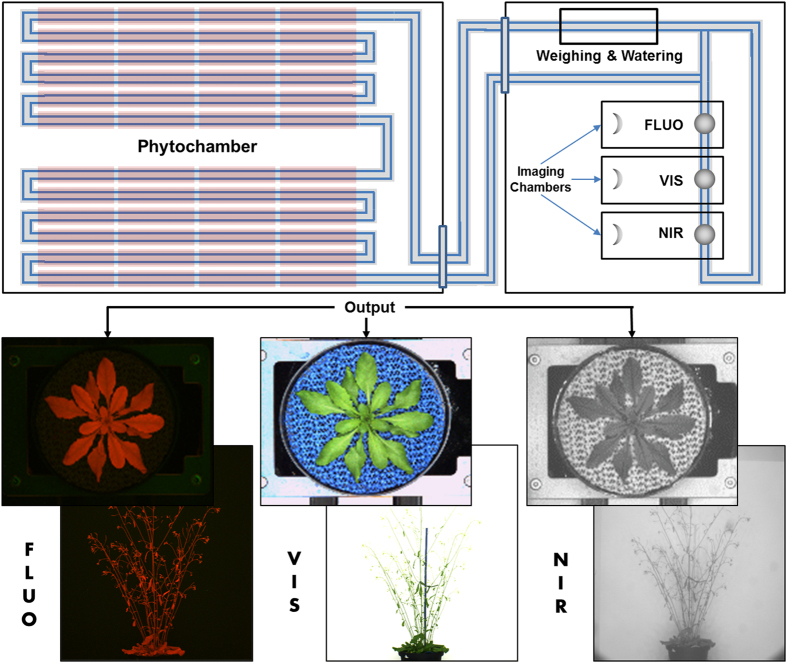
Schematic view of the LemnaTec system for small plants. Top: 384 carriers are situated in 48 blocks of 8 on 12 conveyor belt lanes (6 per side of the phyto-chamber). The carriers with the plants are transported regularly through a tunnel to the imaging chambers and watering/weighing station. A small imaging loop for manual loading of plants into the carriers is connected to the imaging stations and was used here for imaging of the stationary plants. Bottom: Representative images of Arabidopsis plants in rosette stage (top view) and mature plant/flowering stage (side view) which are acquired with the different camera sensors in top and side view.

The presented dataset is derived from the system for small plants (see also ref. 5), such as *Arabidopsis thaliana*, which combines a growth chamber for controlled environmental conditions and the imaging chambers for non-invasive trait assessment. A schematic view of this system is illustrated in Fig. 1. In this study, Arabidopsis plants have been grown with a large number of replicates and their growth and development has been evaluated with respect to the following factors:

1. moving vs. stationary:
2. does the movement on the conveyor belt influence the plant growth?
3. covered vs. uncovered:
4. does the soil cover influence the plant growth (special soil covers are used for reducing transpiration and to facilitate segmentation of plant pixels from the background during image analysis)?

The following datasets have been produced:

1. Raw images acquired by the different camera sensor systems of the LemnaTec system and semantical description of the experimental setup with ISA-Tab^6^ formatted metadata
2. Result images after segmentation and post-processing with result data table containing 310 features after extraction by image analysis

This study addresses fundamental issues of high throughput plant phenotyping experiments and describes the associated multi-stage workflow starting from plant cultivation, over experimental design and image acquisition, image analysis procedures and data publication coupled with standardized metadata representation. The presented workflows using the example of the Arabidopsis phenotyping experiment are used in a similar manner for the LemnaTec facilities for medium sized (e.g. barley) and large plants (e.g. maize) at IPK but are flexible enough to be adapted and reused by scientists of the growing plant phenotyping community. A detailed documentation of the methodology described herein is published in the standardized metadata format ISA-Tab^6^, furthermore integrating the recommendations for *minimal information about plant phenotyping experiments* (MIAPPE)^7^. These have been approved among partners of the *German Plant Phenotyping Network* (DPPN) and the EU-funded *trans-national infrastructure for plant genomic science* (transPLANT) for describing plant phenotyping experiments. We present the first MIAPPE compliant ISA-Tab reference dataset for a high throughput phenotyping experiment which links all metadata, raw and processed data to semantic respectively technical documentations, measurement protocols and references to ontologies.

## Methods

### Plant cultivation

Seeds of the *Arabidopsis thaliana* genotype [C24^8^, NASC ID N22680] have been pre-treated on wet filter paper for one night (20 °C, darkness) before sowing on soil (75% (v) Substrate 1 and 15% (v) sand). In order to initiate germination pots (10cm diameter, with seeds) underwent a stratification period of 3 days at 5 °C in dark. For the germination the environmental conditions in the phyto-chamber were set to long-day conditions (16h day/8h night) at 16/14 °C, 75% relative humidity, and 120 μmol light intensity. After 5 days and for the remaining cultivation period seedlings/plants were grown at identical conditions except for a higher temperature (20/18 °C).

In addition to the 384 pots that were placed in the LemnaTec carriers, 100 pots were placed on tables in the same phyto-chamber (factor: moving/stationary). The soil surface of half of the pots of each condition (factor: moving/stationary) was either left uncovered or was covered with a blue rubber mat (factor: uncovered/covered).

### Image acquisition

All images were automatically acquired using the LemnaTec Scanalyzer system. From 12 *days after sowing* (das) on all plants (factor: moving) have been imaged daily from the top view using all three imaging procedures (VIS, NIR, FLUO). VIS images were acquired in the visible light spectrum (~390–750 nm) using a Basler (Basler AG, Ahrensburg, Germany) Pilot piA2400-17gc (RGB) camera with a resolution of 2056×2454 (side) and 2454×2056 (top) pixels. A fluorescence imaging system (FLUO, excitation: 400-500 nm, emission: 520–750 nm) using a Basler Scout scA1400-17gc (RGB) camera with a resolution of 1234×1624 (side) and 1624×1234 (top) pixels, allowed for the quantification of static fluorescence signals of plants. Near-infrared imaging (NIR) was performed in the wavelength range between 1450–1550 nm using a Nir 300 PGE (Allied Vision Technologies GmbH former VDS Vosskühler GmbH, Stadtroda, Germany) (monochrome camera) sensor with a resolution of side: 254×320 (side) and 320×254 (top) pixels. During the experiment two different zoom configuration for the top-view images were used to cover the maximum imaging area at different plant developmental stages (change at 48 das). Additionally, from 48 das side images were acquired (angles: 0 and 90°).

Stationary plants have been imaged every second day using the small imaging loop with manual loading of the pots/carriers onto the system. Blank reference images (background images without carrier and plants), for each imaging system, were created before each imaging run. All images were saved as uncompressed .png (portable network graphic) files (see Data Record 1).

### Manual trait acquisition

Plants were harvested at 55 das and dry weight determination was performed on a single plant level by cutting the shoot part of each, transferring it into a paper bag and drying it for 3 days at 80 °C. Weighing was done using a fine-scale balance. Plant height was measured at 49 das using a ruler detecting the maximal extension between soil level and the tips of the shoots.

### Image analysis

The image analysis was performed by using the *Integrated Analysis Platform* (IAP) software^9^. IAP is a framework for high-throughput plant image analysis supporting different species and imaging systems. The different steps of the image analysis pipeline can be classified into the following sections: image acquisition, pre-processing, feature extraction and post-processing. An overview of the image analysis pipeline configurations and the image analysis blocks used in the present study is given in Table 1. Parameter modifications were done according to the experiment configuration and imaging conditions.

### Extracted Features

During image analysis 310 features were extracted which can mainly be classified into geometric and color-related traits (including VIS-related 139, FLUO-related 152 and NIR-related 17 features). A complete overview and detailed description can be found in the IAP documentation (https://github.com/OpenImageAnalysisGroup/IAP) and ref. 9. The features are computed for each side and top view image derived from the different camera sensors. Table 2 summarizes traits which were used for data validation by correlation with the manually acquired data (see Technical Validation). Since the presented experiment assessed growth differences under moving/stationary and covered/uncovered conditions the presented analysis focussed on biomass-related geometrical features. All traits have the same naming scheme, e.g.:


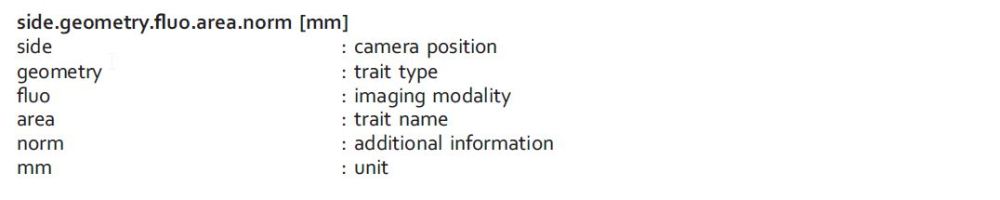


For some geometry-related traits a pixel-to-mm conversion is routinely performed by using the pot and carrier as reference objects with known dimensions.

(1)VLT=At × As.0° × As.90°

*A*_*t*_=computed top area

*A*_*s.0°*_=computed side area (0 degree)

*A*_*s.90°*_=computed side area (90 degree)

### Statistical analyses

Manually collected data (plant height and dry weight) was corrected for outliers based on ±2.5 standard deviation of the mean value using the software package GenStat 16th Edition. For imaging-derived plant features the same method is implemented in IAP and outliers automatically highlighted in the result file. For the correlation analysis and plotting of the corresponding figures the software package SigmaPlot 11.0 was used. Each correlation coefficient, performed as Pearson product-moment correlation, was set with a significance level of *P*<0.001. ANOVA and post-hoc Bonferroni test were performed using GenStat 16th Edition (http://www.vsni.co.uk/software/genstat).

### Code availability

#### Image analysis software

The IAP software is an open-source project and is available at https://github.com/OpenImageAnalysisGroup/IAP. For the presented analysis version 2.0.2 was used.

#### Data storage and publication system

All datasets described in this article are stored in the Plant Genomics and Phenomics Research Data Repository (PGP)^10^, which is hosted at the Leibniz Institute of Plant Genetics and Crop Plant Research (IPK) Gatersleben. The Repository is based on the e!DAL research data storage and publication system^11^, which is available at https://edal.ipk-gatersleben.de. Data records can be accessed via Digital Object Identifiers (DOIs).

## Data Records

### Data Record 1

This dataset (Data Citation 1: Junker et al. *IPK Gatersleben*, http://dx.doi.org/10.5447/IPK/2016/7) consists of the raw image files in the ‘1135FA_images’ folder. The subfolders are ordered and categorized into ‘camera_sensor’ (vis/fluo/nir), ‘camera_view’ (top/side) and ‘das’ (day after sowing). The blank reference images are located in separate folders called ‘null_images’. The dataset is semantically described using the ISA-Tab format^6^. The corresponding files are located in the ‘metadata’ folder. Table 3 shows an overview about the composition of the image dataset.

All images have the same naming scheme, e.g.:


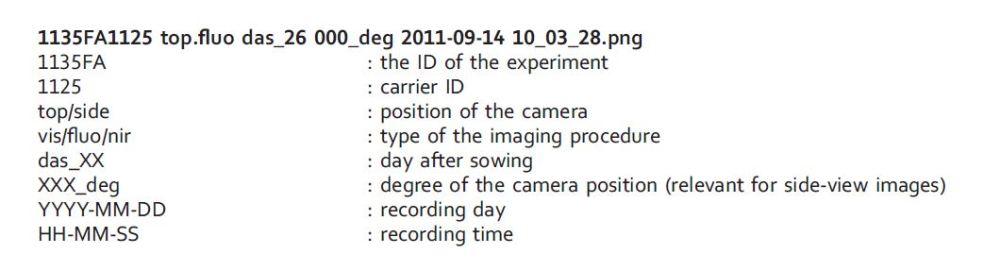


The raw image dataset contains about 10142 raw images as well as 48 reference images acquired with each of the three camera sensor systems. The imaging period started at the 12th das. Until 47th das, covering the period of rosette development, only top view images have been taken. In order to assess plant height after bolting (shoot formation) side view imaging (0 and 90 degree) was started at 48th das. Usually there should be at least 384 images from the non-stationary plants for every imaging day. Missing images are due to technical problems with the phenotyping facility or in rare cases specifically with the camera sensors creating corrupt or one-pixel images (see Technical Validation).

Furthermore the record contains an executable Java-based script ‘convert_to_IAP.jar’ that associates the metadata information with the raw image files and rearranges the raw images into an IAP compatible folder structure to enable the re-analysis. The provided ‘readme.txt’ describe how to run the script (see Usage Notes).

### Data Record 2

This dataset (Data Citation 2: Junker et al. *IPK Gatersleben*, http://dx.doi.org/10.5447/IPK/2016/8) contains all data from the validation experiment (for details see Technical Validation). It includes 1100 raw and 1100 corresponding result image files which are stored in an IAP dataset (1613AJ_IAP_dataset). The image analysis was performed with the IAP software by using the same analysis pipeline configuration as used for Data Record 1. Furthermore the dataset contains a folder ‘animations’ with two *.mp4 videos generated from the series of top (1613AJ_flowering_plant_top_view.mp4) and side view (1613AJ_flowering_plant_side_view_315_deg.mp4) images acquired from the flowering plant which gives a visual impression of the validation experiment and supports the understanding of the extracted results.

### Data Record 3

This dataset (Data Citation 3: Junker et al. *IPK Gatersleben*, http://dx.doi.org/10.5447/IPK/2016/9) comprises 29724 result image files after image analysis with IAP (see Methods). All files are sorted using the same folder structure as in Data Record 1. For every raw image file one corresponding result image was produced. Due to corrupt or missing raw image files a number of 558 result images missing. The file naming scheme is identical to the raw image files (see Data Record 1). Furthermore this record contains the result report from the image analysis including all computed plant features in a tabular format.

## Technical Validation

Validation of the high throughput imaging workflow included procedures for the correction of two levels of potential errors: 1) systematic errors which are related to the general measurement and 2) analysis procedure and random errors, which are related to an individual measurement.

### Systematic errors

The deduction of plant growth profiles from high throughput phenotyping (represented by repeated imaging of the plant throughout the developmental time course of interest) depends on standardized imaging and image analysis procedures which are optimised towards the most exact determination of plant measures. Systematic errors of the established imaging and analysis procedures have been assessed in frame of a validation experiment and by comparison of imaging derived data with manually measured ground truth data.

### Validation experiment

For validation of the imaging and image analysis procedures an experiment has been performed with three Arabidopsis plants (C24 accession) in different developmental stages (early rosette 34 das, late rosette 49 das and one plant in the early flowering stage 72 das) that have been subjected to repeated imaging for assessing the variation in phenotypic features extracted from images taken over 50 rounds from the same objects and with the same configurations. Table 4 summarizes all relevant information about the setup of this validation experiment and Table 5 lists all statistical parameters for the evaluated phenotypic traits.

**Figure 2:**
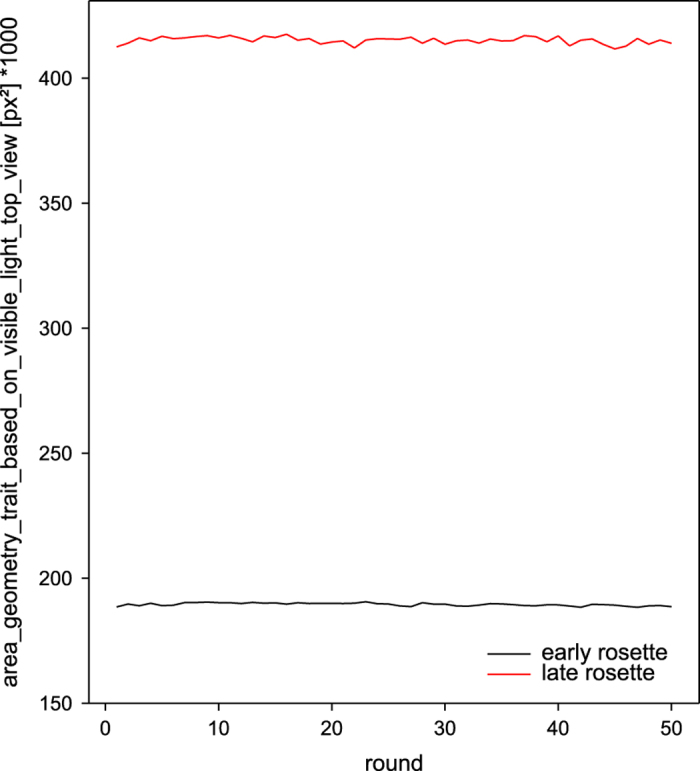
Validation of top view imaging and image analysis procedures. Projected leaf area (area_geometry_trait_based_on_visible_light_side_view [px^2^]) of two Arabidopsis C24 rosette stage plants over 50 rounds of imaging in a high throughput plant phenotyping system. Early rosette (black); late rosette (red).

All images acquired in frame of the validation experiment have been analysed using IAP and extracted geometrical plant features have been statistically evaluated with respect to its variation over rounds of imaging (top and side view images) and over side view angles (side view images). The projected plant (leaf) area values extracted from top view images of the rosette stage plants showed very little variation for the early and late rosette over 50 imaging rounds (coefficient of variation (CV)=0.31 and 0.33 for the early and late rosette, respectively, Fig. 2, Table 5). No side view imaging was performed for rosette stage plants.

**Figure 3:**
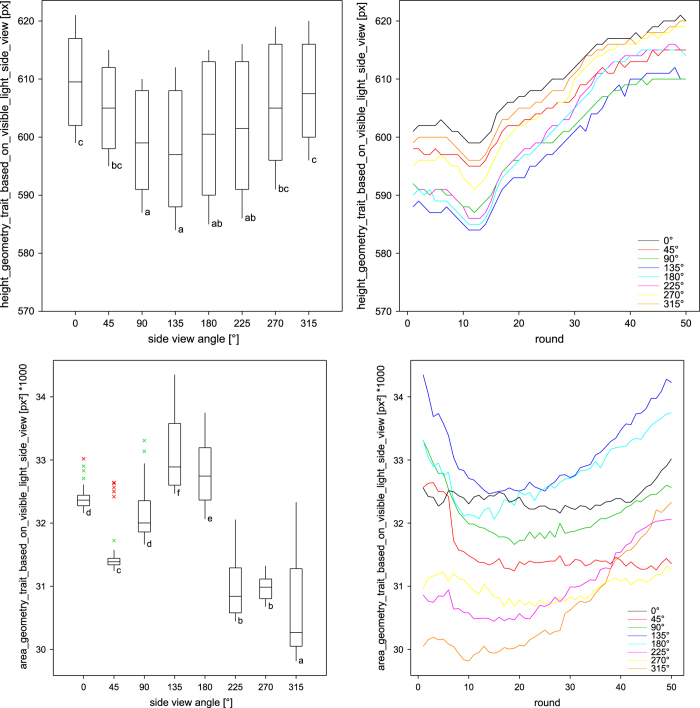
Validation of side view imaging and image analysis procedures. Plant height (height_geometry_trait_based_on_visible_light_side_view [px]) and side view projected leaf area (area_geometry_trait_based_on_visible_light_side_view [px^2^]) of a flowering Arabidopsis plant imaged from 8 different side view angles over 50 imaging rounds. Upper left: Plant height from all rounds plotted over side view angles. Upper right: Plant height for each side view angle plotted over imaging rounds. Lower left: Side view projected leaf area from all rounds plotted over side view angles. Lower right: Side view projected leaf area for each side view angle plotted over imaging rounds. Red and green crosses mark outliers outside the 5 and 2.5% range of the standard deviation of the value distribution, respectively (C). Letters indicate statistically significant differences according to ANOVA and post-hoc Bonferroni test (A, C).

The images of the flowering plant were used for extracting top and side view geometrical features and validating the corresponding imaging and image analysis procedures. For side view imaging a configuration with 8 different side view angles (0, 45, 90, 135, 180, 225, 270 and 315°) was used thereby covering all possible lateral viewpoints on the plant in 45° steps. From the respective images of 50 rounds of imaging plant height [px] and projected side view leaf area [px^2^] were extracted and analysed over time (50 rounds) and side view angle (8 angles). Using the side view angle as factor in an ANOVA reveals small but expectedly significant differences in plant height as well as projected side view leaf area (Fig. 3 upper and lower left).

Changes in plant height are due to the changing orientation of the slightly bent shoot towards or away from the camera sensor in the different angle positions. This is well supported by the course of the change in plant height over all angles which decreases gradually towards 135° and increases in analogous manner towards 0° again with the most similar values extracted from images taken from adjacent side view angles (Fig. 3 upper left). Changes in plant height over time (see Fig. 3 upper right) explains most of the variation observed for the images from the same angle (Fig. 3 upper left) and is due to movements of the shoot as well as plant growth during the imaging time of approximately 1:30 h (see Data Record 2, video 1613AJ_flowering_plant_side_view_315_deg.mp4). Variation of plant height between angles is only marginally dependent on time (see Fig. 3 upper right) thus underpinning the influence of plant orientation (architecture and viewpoint).

Significant changes in projected leaf area from the different side view angles are explained by variation in plant height, additional occlusion effects of different plant parts in the 2D projection and by movements of shoot and leaves which lead to increased or decreased occlusion depending on time and angle (see Fig. 3 lower panel; Data Record 2, video 1613AJ_flowering_plant_side_view_315_deg.mp4). Occlusions occur for example when stem-borne leaves do or do not cover parts of the stem from different viewing angles. Up and down movements of rosette leaves over time lead to smaller or bigger representations in the 2D projection due to increasing and decreasing occlusion by other adjacent rosette leaves. Furthermore movement-induced changes in the orientation of the stem towards or away from the camera sensor explain part of the observed variation over angles and time.

**Figure 4:**
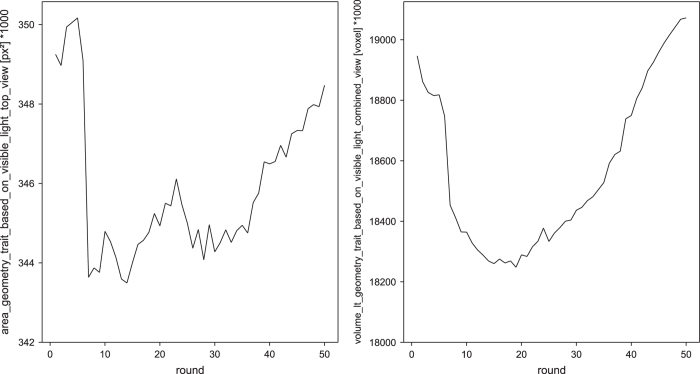
Validation of top view imaging, image analysis procedures and volume estimations for a flowering Arabidopsis plant imaged over 50 imaging rounds. Left: Top view projected leaf area (area_geometry_trait_based_on_visible_light_top_view [px^2^]) plotted over imaging rounds. Right: Volume_LT as an estimate of plant biomass (volume_lt_geometry_trait_based_on_visible_light_combined_view [voxel]) plotted over imaging rounds.

Top view images of the flowering plant have been used for the extraction of the top view projected leaf area (see Fig. 4 left). Variation in top view area is mainly due to movements of the stem (see Data Record 2, video 1613AJ_late_rosette_top_view.mp4). Depending on the orientation of the stem the flower structures are either projected onto the rosette leaves thereby not (completely) being taken into account as additional pixels (smaller area values) or with increasing inclination of the stem apical structures are projected partially or completely ‘outside’ the rosette area and therefore increase the pixel number (higher area values, e.g. in the first rounds of imaging). All variations detected in plant height, side and top view projected areas are cumulated in the trait ‘volume’ (see Figure 4 right) which is calculated according to Equation 1 and represents an estimate of plant biomass. With a CV of 1.45 (see Table 5) the observed variation is acceptably small and reveal that the high throughput imaging and image analysis workflows are performing very stable and with a high resolution.

The evaluation of the validation experiment underlines the high repeatability and reproducibility for the quantification of biomass related geometrical plant features. CV are very small for all traits evaluated (see Table 5). Imaging and image analysis procedures are very sensitive and allow for the detection of short term growth processes. The described variation-inducing effects of plant architecture, orientation (imaging angle) and movement are small but have to be carefully considered by the experimenter and appropriate experimental and imaging procedures are necessary in order to ensure the reliable quantification of plant phenotypic traits^5^.

### Comparison of image derived traits with manually measured ground-truth data

**Figure 5:**
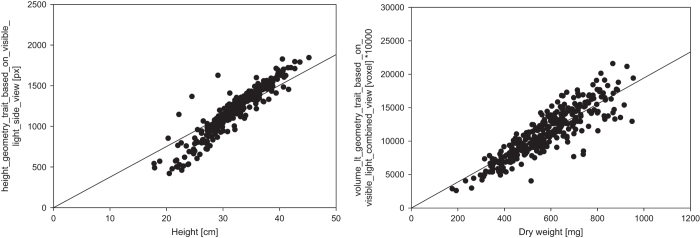
Correlation between manually measured and automatically processed imaging-derived plant features for validation of the phenotyping workflow. Left: correlation plant height (height (geometry trait based on visible-light side view) [px]) at 49th das with manually measured plant height, correlation coefficient r of 0.898 (*P*-value 7.34e^−116^). Right: correlation estimated plant volume (volume prism (geometry trait based on visible-light combined view) [voxel]) at 55th das with manually measured plant dry weight, correlation coefficient r of 0.876 (*P*-value 5.86e^−103^).

The manual assessment of plant traits complements and validates the results derived from automated high throughput plant imaging and image analysis on a systematic level including the functionality of all hard- and software components involved in the process. For the current data descriptor, two traits have been analysed manually on a single plant basis: plant dry weight (invasive measurement at the end of the experiment, 55 das) and plant height (non-invasive measurement close to the end of the experiment, 49 das). Pearson-correlation based comparisons between plant dry weight [mg] and imaging-derived estimated volume [voxel] as well as plant height and imaging-derived plant height revealed high positive correlations with r of 0.876 (*P*-value 5.86e^−103^) and r of 0.898 (*P*-value 7.34e^−116^) (see Figure 5). These results (see Data Record 3) provide an indirect, but clear evidence for a correct mode of operation of all hard- and software components of the phenotyping facility (e.g. controlling and settings of the imaging units) as well as the experiment-specific, properly configured image analysis pipeline in the IAP software.

### Random errors

**Figure 6:**
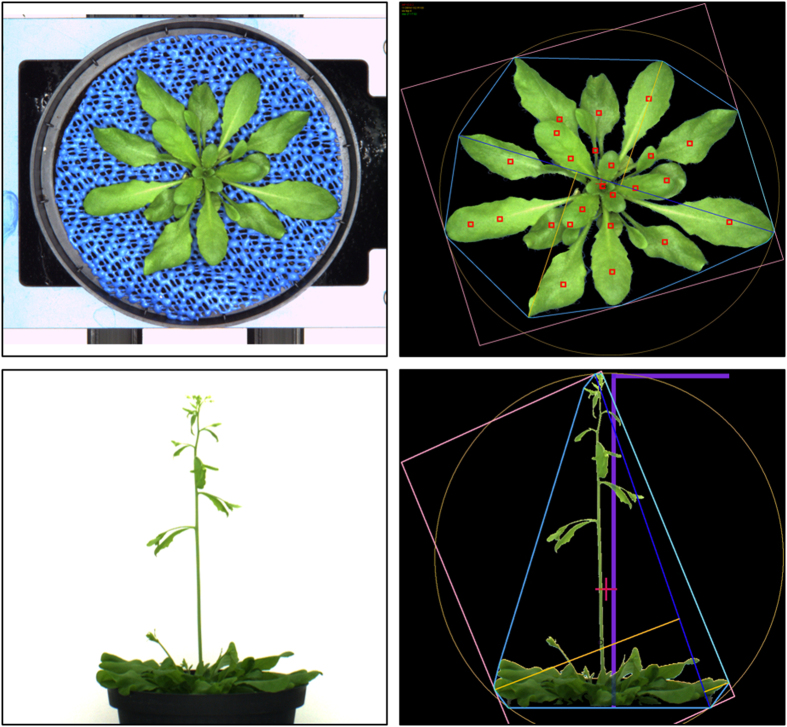
Representative top and side view images acquired with the VIS camera system and respective output images after segmentation and feature extraction (using IAP). Left: top view image of 27th das and the side view image of 38th das for one single plant taken by VIS camera system. Right: visualization of the respective IAP processed images. The computed features are different bounding areas (pink area), leaf axes (dark blue line), leaf centroids (red points) and rosette diameter (orange circle), plant height (purple line) and convex hull (blue area).

Besides systemic influences on the output of a phenomics experiment (general settings of the hardware or/and image analysis pipeline), non-systematic errors can occur occasionally and in an experiment-specific manner. Different automated/semi-automated and manual steps were implemented for correction of these errors. Due to measurement, imaging or camera errors, empty or one pixel images might be generated in the course of an imaging round. These images are automatically detected during image analysis with IAP (see Usage Notes) and not taken into account for any calculation or feature extraction. Furthermore, they will be highlighted in the output image dataset for easy recognition by the experimenter. During the export of the numeric data, for each extracted feature outliers (values < 2.5% and > 97.5% of the value distribution) will be color-coded in the result data table (see Data Record 3) and provide the basis for semi-automatic validation of the image analysis pipeline through cross-validation of values and corresponding result images (Fig. 6 right) by the experimenter. After cross-validation outliers might be easily removed for further post-processing of the data. Finally, manual checking of the result images (Fig. 6 right) and plotting of imaging-derived parameters (such as growth curves) are recommended and supported by IAP. By these means the experimenter is enabled to validate the phenomics workflow before starting data post-processing.

## Usage Notes

All images of the presented datasets have been analysed using the *Integrated Analysis Pipeline* (IAP)^9^. IAP is designed to analyse 2D whole plant image time series derived from VIS, NIR and FLUO imaging with top and side views. It supports the automated extraction of a wide variety of geometrical and plant architectural, color- and texture-related features as well as physiology-related traits for shoot images of different plant species. The IAP image analysis pipelines can be adapted to extract features from images acquired with various sensor systems (independent from LemnaTec), for example images acquired by a scanner or low-cost SLR. Though specific requirements have to be fulfilled with respect to foreground/background coloration and contrast as well as image resolution. Therefore, IAP as an open-source system, allows for the dynamic integration of further algorithms in order to extend the feature spectrum to be extracted. The latest version does not support the analysis of 3D imaging data and hyperspectral data, although current developments are aiming at the integration of novel sensor data. Limitations in image analysis are mostly due to the insufficient quality of the raw images (in terms of resolution), structural problems (organ/region of interest too small), insufficient contrast (features discriminating foreground vs. background or organ of interest vs. whole plant), or currently missing IAP support for image data acquired with specific sensors.

In the following we provide a short tutorial on how to re-analyse the data using the IAP software. All steps of the analysis are also described in the ‘readme.txt’ (see Data Record 1)

1. Create IAP dataset

- make sure that the raw image folder ‘1135FA_images’ and ‘convert_to_IAP.jar’ are located in the same directory
- execute the jar file (‘java -jar convert_to_IAP.jar’), a folder with the IAP dataset named ‘1135FA_IAP’ will be created (a proper Java 1.7+ runtime needed)

2. Start analysis with IAP

- start IAP (version 2.0.2: https://github.com/OpenImageAnalysisGroup/IAP)
- load the dataset by clicking on ‘Load or Create Dataset’ → ‘Load Dataset(s) from File System’ and navigate to the experiment overview screen
- start the analysis by clicking on ‘Analysis’ → ‘Process Arabidopsis Analysis’, image analysis settings might be changed using the ‘Export Modify Settings’ Button (approximate time of the re-analysis: 6 h; Quad-Core CPU, 16GB RAM)
- after the analysis, the result dataset is opened automatically and the following options can be used:

1. Images can be browsed and potential outliers can be detected (‘View Images’)
2. Numeric data can be visualised (‘Create Data Chart’)
3. Images and the numeric data can be exported (‘Export’)

By performing the IAP re-analysis of the provided Data Record 1 one is enabled to further explore the capabilities of this image analysis software especially with regard to other traits besides biomass-related geometrical plant traits. These traits have been in the focus of the present evaluation as the most relevant features when comparing Arabidopsis thaliana growth with the given experimental factors (rotating/stationary and covered/non-covered). Other traits, especially derived from FLUO and NIR images, have been shown to be relevant in different experimental contexts^12–15^.

In general, the dataset can be used as a reference dataset for testing and evaluation of new image analysis methods. The provided manually measured plant features can be used as ground truth data to validate the algorithms for biomass estimations. Furthermore, approaches for calculating architectural features such as leaf counts or individual leaf segmentation can be tested and further methods for sensor fusion and time series analysis might be applied. The provided detailed and standardized documentation of experimental metadata and results, which is conform to the MIAPPE recommendations, guarantees reuse and comparability of the dataset. Given the structural complexity of phenotyping datasets and the connected necessity for metadata documentation, the here presented dataset and procedures have the potential to become applied in a community-wide manner.

## Additional Information

**How to cite this article**: Arend, D. *et al.* Quantitative monitoring of *Arabidopsis thaliana* growth and development using high-throughput plant phenotyping. *Sci. Data* 3:160055 doi: 10.1038/sdata.2016.55 (2016).

## Supplementary Material



## Figures and Tables

**Table 1:** Overview of the used IAP image analysis blocks

**Block name**	**Description**
*Acquisition*	
Load Images	Load different camera images from local hard drive or database
*Pre-processing*	
Color Balancing Vis	Apply vertical color balancing on VIS image
Background Correction Fluo	Apply vertical color balancing on FLUO image
Background Correction Nir	Removes shading from NIR image
Rotate Images	Correct rotation
Align Images	Align position and scale of different camera images
Detect Blue Markers	Calculate factor for scale conversions (pixel → millimetres)
Clear Masks Well Processing	Crop round mask
*Segmentation*	
Adaptive Vis Segmentation (KMeans)	K-means based segmentation on VIS image
Adaptive Segmentation Fluo	Create intensity images for red and yellow reflectance
Median Filter	Apply median filter
Remove Small Noise Objects	Remove artefacts on VIS and FLUO image
Adaptive Threshold Nir	Apply adaptive threshold on NIR image
Apply Fluo Mask to Other	Use mask from FLUO image to clear other images (VIS, NIR)
*Feature Extraction*	
Skeletonize Vis Fluo	Calculate the skeleton by thinning on VIS and FLUO image
Skeletonize Nir	Calculate the skeleton by thinning on NIR image
Calculate Width And Height	Determine plant height and width
Calculate Center of Gravity	Calculate centre of gravity
Calculate Areas	Calculate plant areas (projected area)
Calculate Volume Estimations	Estimate plant volume
Calculate Color- and Intensity- Histograms	Calculate overall color properties (intensity calculation, plant color indices, histogram calculations)
Calculate Convex Hull	Calculate convex hull based shape parameters
Detect Leaf Center Points	Estimation of leaf centres by using a distance map approach
*Post-processing*	
Run Post Processors	Draw analysis results of feature extraction blocks
Move Mask Set to Image Set	Transfer images to result image set
Crop Result Images	Crop result images
Highlight Null Results	Mark errors and outliers in result image set
Save Result Images	Save result images in result data set

**Table 2:** Image processing evaluation—IAP traits used for validation purposes.

**Trait name**	**IAP name**	**Unit**	**Description**
height	side.geometry.vis/fluo.height	px	visible y expansion
volume	combined.geometry.volume.lt	voxel	estimated plant volume (based on approximation, see formula 1)

**Table 3:** Overview of the raw images files.

	**visible (VIS)**	**fluorescence (FLUO)**	**near-infrared (NIR)**
moving plants—top view	6804	6804	6804
moving plants—side view	2568	2568	2568
stationary plants—top view	316	316	316
stationary plants—side view	406	406	406
reference images—top view	30	30	30
reference images—side view	18	18	18
total number of raw images	10142	10142	10142
The dataset contains 30426 raw image files from 384 moving plant and 100 stationary plants.			

**Table 4:** Overview of the setup of the validation experiment

**plant stage**	**genotype**	**imaging modes**	**imaging rounds**	**total imaging time (h:min:sec)**	**evaluated traits**
early rosette	C24	top view, VIS/ FLUO	50	01:25:18	area (geometry trait based on visible-light top view) [px^2^]
late rosette	C24	top view, VIS/ FLUO	50	01:25:18	area (geometry trait based on visible-light top view) [px^2^]
flowering plant	C24	top/side view (8 angles), VIS/ FLUO	50	02:32:45	height (geometry trait based on visible-light side view) [px]; volume lt (geometry trait based on visible-light combined view) [voxel]; area (geometry trait based on visible-light top view) [px^2^]; area (geometry trait based on visible-light side view) [px^2^]

**Table 5:** Summary statistics for the phenotypic traits evaluated in frame of the validation experiment.

**plant stage**	**early rosette**	**late rosette**	**flowering plant**			
**phenotypic trait**	**area (geometry trait based on visible-light top view) [px^2^]**	**area (geometry trait based on visible-light top view) [px^2^]**	**height (geometry trait based on visible-light side view) [px]**	**area (geometry trait based on visible-light side view) [px^2^]**	**area (geometry trait based on visible-light top view) [px^2^]**	**volume lt (geometry trait based on visible-light combined view) [voxel]**
Observations	50	50	400	400	50	50
Mean	189,543	415,143	604	31,824	345,887	18,568,109
Median	189,658	415,250	604	31,866	345,122	18,460,631
Minimum	188,403	411,694	584	29,813	343,496	18,248,135
Maximum	190,611	417,549	621	34,350	350,167	19,072,169
Range	2,208	5,855	37	4,537	6,671	824,034
Standard deviation (SD)	590	1,387	10	931	1,863	269,142
**Coefficient of variation (CV)**	**0.31**	**0.33**	**1.60**	**2.92**	**0.54**	**1.45**
